# A modified behavioral test protocol for simultaneously evaluating the incentive value of alcohol drinking and social interaction in mice

**DOI:** 10.1016/j.mex.2023.102267

**Published:** 2023-06-21

**Authors:** Haodong Su, Ye He, Yuying Li, Kangguang Lin, Guiyun Xu, Tifei Yuan, Zhe Shi, Chunlu Li

**Affiliations:** a School of Humanities and Social Sciences, Binzhou Medical University, Yantai, China; b The Affiliated Brain Hospital of Guangzhou Medical University, (Guangzhou Hu-iai Hospital), Guangzhou Medical University, Guangzhou, China; c Shanghai Key Laboratory of Psychotic Disorders, Shanghai Mental Health Center, Shanghai JiaoTong University School of Medicine, Shanghai, China; d Co-innovation Center of Neuroregeneration, Nantong University, Nantong, China; e Key Laboratory for Quality Evaluation of Bulk Herbs of Hunan Province, Hunan University of Chinese Medicine, Changsha, Hunan 410208, China; f Department of Psychology, School of Medical Humanitarians, Guizhou Medical University, Guiyang, China

**Keywords:** Social interaction, Alcohol preference, Protocol, A modified behavioral test protocol for simultaneously evaluating the incentive value of alcohol drinking and social interaction in mice

## Abstract

Individual sociability and alcohol drinking are interacted to escalate alcohol use. An impairment in perceiving and discriminating the difference in incentive values between social interaction and drinking behavior indicates a shift from moderate alcohol consumption to misuse. However, few studies have evaluated the incentive value of these two behaviors in the same scenario. Thus, we modified a behavioral test protocol to evaluate rodents’ ability to perceive and discriminate the differences in incentive value between alcohol drinking and interaction with their social partners. The present protocol is simple and practicable. Only 2–3 days are required to complete the whole process. Compared with existing methods, our protocol is simple and practicable. Our findings suggested that subtle changes in the incentive value of distinct behaviors can be accurately and reliably assessed using the present protocol in mice with low or high levels of alcohol preference.

• We described a modified behavioral test protocol to simultaneously evaluate the incentive value of alcohol drinking and social interaction.
• The subtle changes in the incentive value of mice with different levels of alcohol preference can be accurately and reliably assessed in the present protocol.
• Using our modified protocol, the differences of incentive value between distinct behaviors can be accurately and reliably assessed in mice with different risks to develop into AUD.

## Graphical abstract

**Figure:**
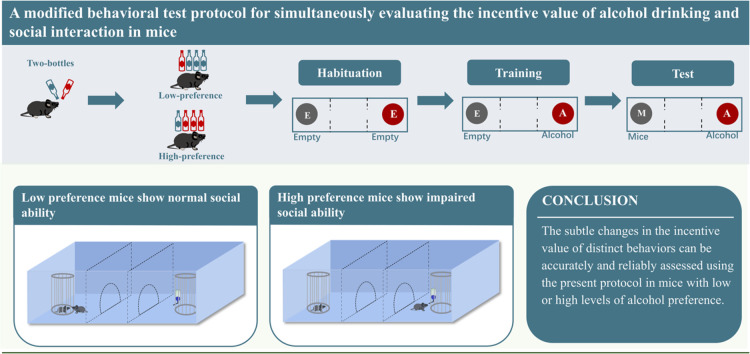

Specifications table

**Table:** 

Subject area:	Neuroscience
More specific subject area:	Animal Behavioral Sciences
Name of your method:	A modified behavioral test protocol for simultaneously evaluating the incentive value of alcohol drinking and social interaction in mice
Name and reference of original method:	No
Resource availability:	• Four three-chamber apparatus were set in a quiet room. • Drinking bottles were fixed to the same height in each cylindrical cage that located in the same chamber of each apparatus (see Fig. 1(b)). Each chamber measures 200 mm (length) × 400 mm (width) × 220 mm (height). **Fig. 1:** 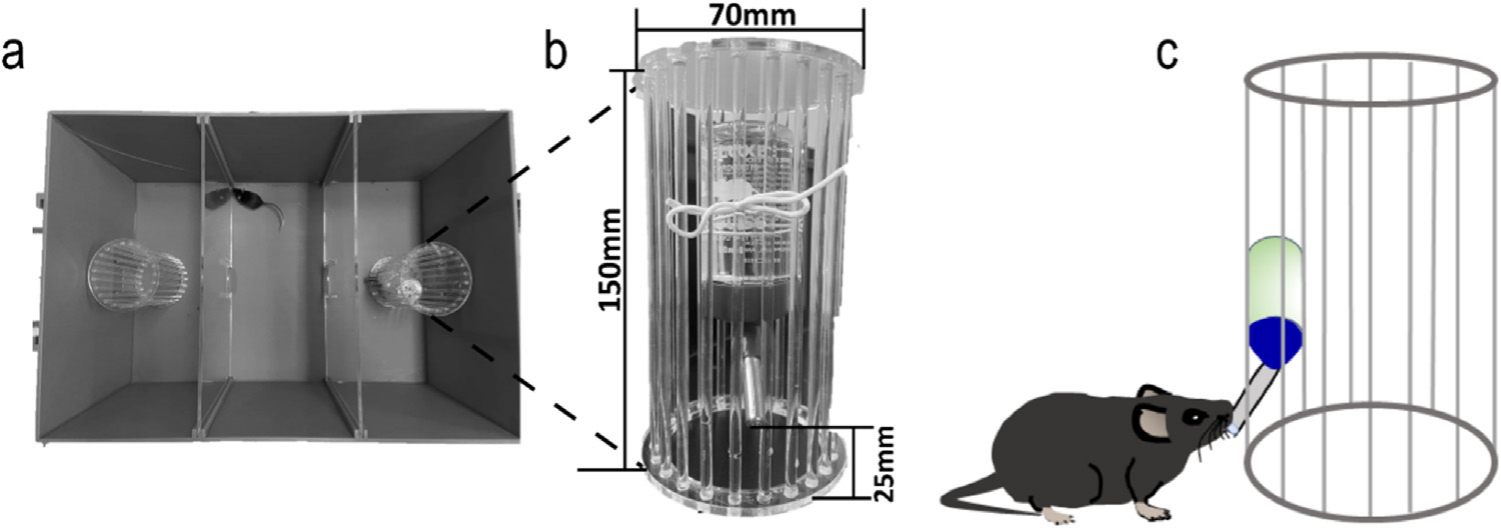 Schematic representation of modified three-chamber apparatus. a Top view of modified three-chambers apparatus; b Illustration of detailed modifications of drinking bottle fixed to the cylindrical cage; c Illustration of mice drinking in the experiment. • Standard three-chamber apparatus: purchased from Shanghai JiLiang Software Technology Co., Ltd. (Model: JLBehv) (Fig. 2). **Fig. 2:** 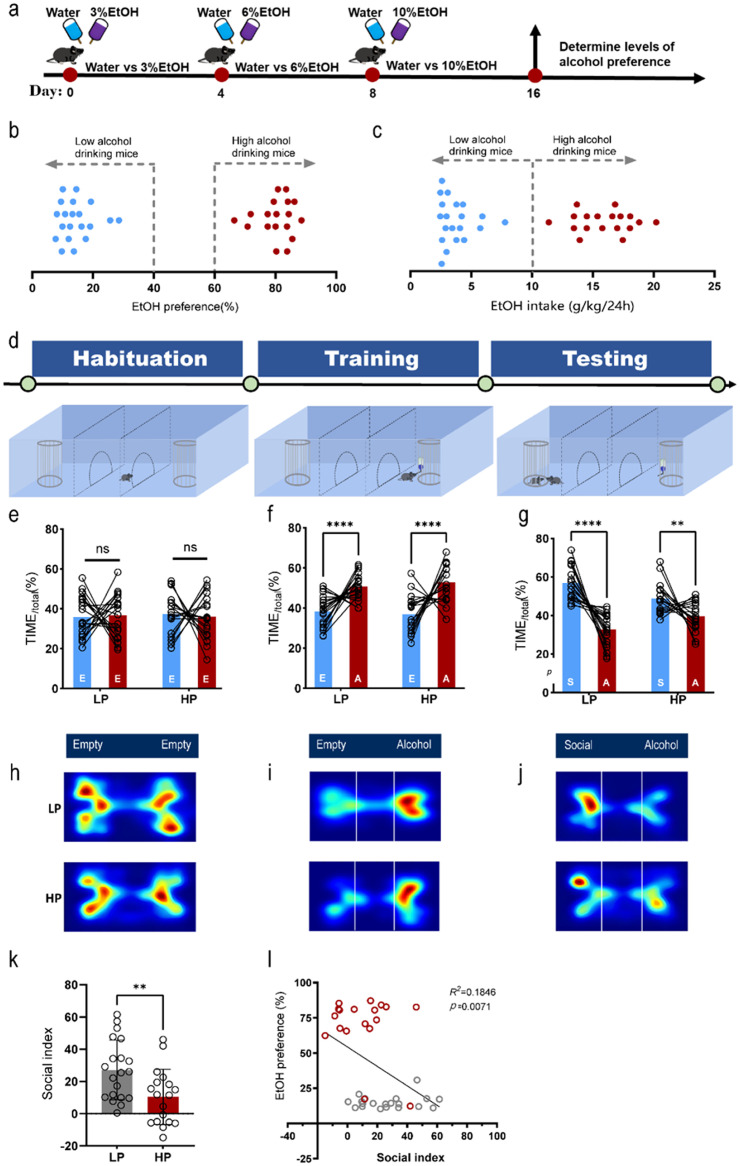 High drinking preference mice showed significantly impaired sociability. a Experimental scheme of a 16-day two-bottle choice alcohol drinking paradigm; b Determine low and high alcohol preference by preference ratio; c Determine low and high alcohol preference by EtOH consumption over 24 h; d Schematic diagram of our modified behavioral test protocol; e Apparatus bias of low and high alcohol preference mice in the habituation phase (no obvious apparatus bias was observed in both HP (n = 18) and LP (n = 20) groups (Two-way ANOVA, Position effect F(1,72) =0.05724, *p* = 0.8116; Empty vs Empty post hoc Bonferroni's multiple comparisons test, LP: *p*>0.9999, HP: *p*>0.9999)); f Choice preference between empty cage and alcohol drinking in the training phase (both HP (n = 18) and LP (n = 20) mice showed a significant preference for alcohol drinking (Two-way ANOVA, Position effect F(1,72)=56.46, *p*<0.0001; Empty vs 10% Alcohol post hoc Bonferroni's multiple comparisons test, LP: *p*<0.0001, HP: *p*<0.0001); g Choice preference between social interaction and alcohol drinking in the testing phase (both HP (n = 18) and LP (n = 20) mice showed a significant preference for their social peer (Two-way ANOVA, Position effect F(1,72)=78.75, *p*<0.0001; Social vs Alcohol post hoc Bonferroni's multiple comparisons tests, LP: *p*<0.0001, HP: *p* = 0.0025); h-j Representative heat maps of position probability of the test mice in the habituation, training and testing phases; k HP mice showed significant impairment in sociability (Unpaired two-tailed t-test, *p* = 0.0066); l Alterations in social index were closely correlated with levels of alcohol preference (R^2^= 0.1846, *p* = 0.0071. n = 38). • Drinking water bottle: Polypropylene. (80 ml; Model: qw2201108). (see Fig. 1b). • Camera: HIKVISION (Model:DS-2CD7A447FWD-XZ(S)(/JM)(/PTZJ)(B). • Video capture card: purchased from Aver Media (Model: GC311) • Anhydrous ethanol: 10% (vol/vol) ethanol diluted in double distilled water. • 75% ethanol was used to clean test instruments and objects between tests. Alcohol is purchased from the National Pharmaceutical Group Chemical Reagent Co., Ltd. these reagents are also available from other suppliers. (Reagent code: 10,009,259; specification: GR (Hushi, ≥ 99.8%)). • Pure water: use laboratory-made pure water. • Alcohol pressure spray can for cleaning. • A transparent glass rod is used to drive away mice. • Nylon short rope for fixing alcohol bottles. • Cotton cloth for wiping. • Graduated Cylinder: Prepare 75% alcohol, 5000 ml, and 250 ml. • Plastic bag: dedicated for laboratory medical waste.

**Method details**

## Materials and methods

### Animals

Male C57BL/6 (8 weeks old) mice weighed 14 – 21 g were single housed in standard mouse cages with corncob bedding and nesting material. Mice were kept in a room with a 12 h light cycle (7:00–19:00) and kept at 22±2 °C. They were provided with standard chow ad libitum.

### Experimental design

The experimental scheme was shown in Fig. 2(d). The whole process contains four stages: (1) Preparation; (2) Habituation; (3) Training; (4) Test.

In the preparation phase, test mice were subjected to a two-bottle choice paradigm to determine the levels of alcohol preference. Under this paradigm, mice can voluntarily take alcohol and develop a stable level of preference [1]. Novel mice were used as social stimuli. It must be ensured that all novel mice behave in a consistent and docile manner. Novel mice were matched with the test mice in age, sex, weight and breed. The aim of this phase was to reduce stress and anxiety when novel mice were placed in the cylindrical cage located in the test apparatus. In general, it takes the novel mice two days to acclimate the environment in the test chamber.

The habituation stage was used to minimize stress responses when high and low alcohol preference mice were placed in the empty apparatus, and to determine whether they showed apparatus bias. The training stage was designed to train the alcohol drinking mice to freely access to alcohol in the apparatus. To avoid apparatus bias, alcohol containing bottle was randomly appeared on one side of the apparatus. In the testing stage, the discrimination and choice between drinking and social interaction in mice were tested in the same scenario.

## Procedure

### Two-bottles choice

After one week acclimation, the test mice were single housed and subjected to continuous access (24 h) to two bottles containing either alcohol or tap water to determine their alcohol preference levels (see Fig. 2a). On day 16, alcohol preference levels of subject mice were calculated. Subsequently, a preference test between alcohol drinking and social interaction was conducted.

### Preparation

Step1: Environmental acclimation. Novel mice (social stimulus) were placed in the testing room for 1 h in their homecage to acclimate to the ambient environment. Step2: Acclimation of the apparatus. After step 1, novel mice were placed in the cylindrical cage for 10 min to acclimate to the internal environment of the test apparatus. Disruptive behaviors, such as bar-biting, excessive self-grooming, circling, or clinging to the sidebars with all four paws could gradually be reduced in most mice [2]. Mice displayed any abnormal behaviors after 10 min acclimation, should be excluded from the experiment. Step3: General behaviors of mice were video recorded and analyzed with software. Mice employed as social stimulus must behave normally.

### Habituation

Step4: Environmental acclimation. Test mice (low and high alcohol preference mice) were placed in the testing room for 60 min in their homecage to acclimate to the ambient environment. Step5: Acclimation of the apparatus. Test mice were gently placed in the middle chamber, and allowed to enter and explore the three-chamber apparatus for 10 min. The exploratory activity was recorded by an overhead camera. Mice were returned to their homecages when the acclimation was finished. Wipe the floors of the chambers, cylindrical cage, and bottles with 75% (vol/vol) ethanol to remove any residual odors [3].

Apparatus bias = (*T*_1_/ *T*_total_) × 100%. Apparatus bias is defined as the preference for one chamber over the other. It can be reflected by the time ratio stayed in each chamber. *T*_1_, time spend in one chamber; *T*_total_, total test time (600 s).

### Training

Step6: Test mice were firstly kept in the middle chamber with gates closed. Then, an alcohol-containing drinking bottle was fixed to one cylindrical cage and left the other one empty. It takes about 5 min to complete. Step7: Subsequently, the left and right gates of the middle chamber were open. Test mice were allowed to explore the three chambers and free access to alcohol for 10 min. The drinking bottle can be easily found. Drinking in the chamber can be easily learned as well. The drinking behavior was recorded by an overhead camera. Mice were returned to their homecages when the training was finished. Then, wipe the floors of the chambers, cylindrical cage, and bottles with 75% (vol/vol) ethanol to remove any residual odors.

### Testing

Step 8: Test mice were firstly kept in the middle chamber with gates closed. Then, an alcohol-containing drinking bottle was fixed to one cylindrical cage and one novel mouse was placed in the other one. It takes about 5 min to complete. Step 9: Subsequently, the left and right gates of the middle chamber were opened. Test mice were allowed to explore the three chambers and free access to either alcohol or social peer for 10 min. Behaviors were recorded by an overhead camera. Mice were returned to their homecages when the training was finished. Then, wipe the floors of the chambers, cylindrical cage, and bottles with 75% (vol/vol) ethanol to remove any residual odors. Step 10: When test was finished, apparatus were thoroughly cleaned. It takes about 3 min [4].

Choice preference (Social interaction and Alcohol preference) and social index. Preference(S)=(*T*_S_/*T*_total_) × 100%; Preference(A)=(*T*_A_/*T*_total_) × 100%; Social index=(*T*_S_-*T*_A_)/(*T*_S_+*T*_A_) × 100%. *T*_S_, time stayed in the chamber where social peer was presented; *T*_A_, time stayed in chamber where alcohol containing bottle was presented; *T*_total_, total test time. The social index reflects the sociability of the test mice.

### Behavior analysis software

A behavior analysis system purchased from Shanghai Jiliang Software Technology Company was used for video tracking and analysis. This video analysis system employs grayscale analysis to identify the movement of mice, with experimental parameters including recognition range and threshold being set. In this experiment, the recognition range was set to be greater than or equal to 50 pixels, while the threshold range was set between 25 and 75. In different laboratory environments, this parameter can be adjusted appropriately to achieve better recognition results. Social behaviors analysis includes distances, movement durations, and contacting times and durations [5]. Behavior was defined as social interaction when the head of the experimental mice was facing the social mice and the distance was not greater than 1 cm [4].

### Statistics

GraphPad Prism 9.3.0 software (GraphPad Software Inc.) was used for statistical analysis. Parametric analyses such as ANOVA (RM two-way and one-way) and unpaired t-test (two-sided) were conducted for data. If the dataset did not have equal variance, repeated-measures ANOVA with the Geisser-Green house correction were used. For ANOVA analyses, Bonferroni's multiple comparison tests were used as post hoc test. *P*<0.05 was accepted as statistically significant. All data were represented as Mean + SD.

## Data for validating the method

### Mice were divided into low or high alcohol drinking group

We utilized a continuous access (24 h), 16-day two-bottle choice alcohol drinking paradigm to generate individual low and high alcohol drinking mice (Fig. 2a). Alcohol preference ratio and consumption over 24 h were used to determine low and high alcohol preference (Fig. 2b). Low alcohol drinking mice were defined as having both EtOH preference below 40% and EtOH intake below 10 g/ kg per 24 h. High alcohol drinking mice were defined as having an EtOH preference of more than 60% and EtOH intake of more than 10 g EtOH/kg per 24 h. EtOH preference and intake remained consistent across the alcohol drinking paradigm (Supplemental Fig. 1a, b).

### The sociability of high drinking preference mice is impaired

As shown in Fig. 2(e), both alcohol low-preference (LP) and high-preference (HP) mice did not show obvious apparatus bias in the habituation phase. In the training phase when mice were trained to freely access to alcohol within the apparatus. As demonstrated by the preference ratio (time spent in either alcohol presented or empty chamber), mice in both LP and HP groups showed significant preference for alcohol drinking (see Fig. 2(f)).

Subsequently, social partners and alcohol were simultaneously presented in the test phase. Although both LP and HP mice preferred to communicate with their social partners, as expected, we noticed that HP mice displayed a significant reduction in social index (see Fig. 2(g) and (k)). We further performed a linear regression analysis on the correlation between social index and levels of alcohol preference. As shown in Fig. 2(l), a significant negative correlation was observed between them.

## Ethics statements

All procedures were proved by the Institutional Animal Care and Use Committee at the Hunan Animal Experimental Center and conformed to the National Institutes of Health Guide for the Care and Use of Laboratory Animals.

## CRediT authorship contribution statement

**Haodong Su:** Formal analysis, Investigation, Writing – original draft, Visualization. **Ye He:** Formal analysis, Investigation, Writing – original draft, Visualization. **Yuying Li:** Formal analysis, Investigation, Writing – original draft, Visualization. **Kangguang Lin:** Supervision, Writing – review & editing. **Guiyun Xu:** Supervision, Writing – review & editing. **Tifei Yuan:** Conceptualization, Methodology, Supervision, Project administration, Writing – review & editing. **Zhe Shi:** Conceptualization, Methodology, Supervision, Project administration, Writing – review & editing. **Chunlu Li:** Conceptualization, Methodology, Supervision, Project administration, Writing – review & editing.

## Declaration of Competing Interest

The authors declare that they have no known competing financial interests or personal relationships that could have appeared to influence the work reported in this paper.

## Data Availability

Data will be made available on request.
